# "The Hospital" Nursing Mirror

**Published:** 1897-11-20

**Authors:** 


"The Hospital" Nursing Mirror.

Being the Nursing Section of "The Hospital."

[Contributions for this Section of "The Hospital" should be addressed to the Editor, The Hospital, 28 & 29, Southampton Street, Strand, London, W.C., and should have the word "Nursing" plainly written in left-hand top corner of the envelope.]

### News from the Nursing World.

### IN MEMORIAM.

A gift of £100 has been made by Mr. C. F. Cory Wright to the Great Northern Central Hospital, Holloway Road, N., to open a fund to endow a bed in memory of H.R.H. the Duchess of Teck. Donations to complete the fund may be sent to the Baroness Burdett-Coutts, or to the hon. secretary of the Ladies' Association, Mrs. Harkness, at the hospital.

### BROMPTON CONSUMPTION HOSPITAL.

A journalist who is interested in hospitals and desirous of helping them called at this hospital recently, and sends the following account of what was uttered by a responsible but reticent official. It is a first qualification for successful service in a voluntary hospital nowadays to possess the faculty of welcoming and trusting the press, a fact not always appreciated as it should be by the authorities, as the following account shows:—"The nursing at Brompton Consumption Hospital must be unusually monotonous if we may form an opinion from the answers we received to our enquiries. We were told for instance that 'nothing worthy of note has happened lately. The lady superintendent is away on the remaining portion of her summer holiday, and her second in command is fulfilling her duties. The beds are full, but the subscription lists are not, this hospital unfortunately being one of those which has suffered (?) in consequence of the Prince of Wales's Hospital Fund. Nevertheless, a special effort is being made to obtain money to build a convalescent home, as consumptive patients are not now acceptable nor eligible guests at general convalescent homes. Three things only are known as yet with regard to the new home—First, it is to be called after Her Majesty; secondly, it is to be built in separate cottages; and, thirdly, that a sum of £20,000 will at least be required, of which about £1,000 has been subscribed.'" It is surprising to be told that the Brompton Hospital has suffered from the Prince of Wales's Fund, which we beg leave to doubt, seeing that we learn that the best-managed hospitals in London were never more prosperous. Ill-considered statements of this kind, which the annual accounts may not confirm, should not be circulated, as they may, like curses, come home to roost.

### TRAINED NURSES' ANNUITY FUND.

The committee of this fund is anxious to raise £1,000 in order to increase the pension granted to superannuated nurses from £15 to £18 per annum. Twelve annuities are granted by this society, and its work is valuable to those nurses who have not been able to avail themselves, from one cause or another, of the pension fund. There are many nurses who bore the heat and burden of the day before pensions and certificates were on everyone's lips, and they are entitled to support and help in sickness and age to which their more fortunate successors have no claim.

### THE SCHOOL BOARD ELECTION.

We are glad to learn that Miss V. Honnor Morten, whose address we have received, is a candidate at the present School Board Election. Miss Honnor Morten was for some years a member of The Hospital staff. Her knowledge of nursing matters and hygiene, and her interest in the condition of those less fortunately situated than herself, have been already practically demonstrated. Women representatives have been markedly successful on School Boards in the past, and we trust that on the present occasion the electors of Hackney will return Miss V. Honnor Morten as one of their representatives.

### A NURSE'S CAUSE.

Amongst the candidates for a pension from the British Home for Incurables is Ellen Hilton Cooper, aged 54. She is a trained nurse, and was at one time the owner of a profitable nurses' home. She married early, and her husband dying suddenly of heart disease within three weeks of her wedding day, she went to St. Thomas's, and there trained as a nurse. The cause of her present distress is the failure of health, which necessitated the management of her home passing into other hands which proved unscrupulous. She is now dependent for a home on the daughter of a former patient, herself in straitened circumstances. She now suffers from angina pectoris and other internal troubles; and has no money, no relatives, and no home except that provided by the charity of one who is herself poor. Miss Dover, 55, Buckingham Road, Aylesbury, who is working on her behalf, will gladly give full particulars of the case.

### BATH CHAIRS FOR BATH.

The medicinal springs at Bath have been in repute since the early days of the Roman occupation of Britain. Lately efforts have been made to attract once more the tide of invalids which fashion had turned in other directions; therefore the following incident, showing how keenly alive the railway company is to the progress of the city and also to its own earnings, offers a significant comment on the lack of enterprise that occasionally characterises our fellow countrymen. A lady, crippled with rheumatism, resolved on a pilgrimage from Yorkshire to the healing waters of the celebrated spring. In every change she was supplied by the porters with an invalid chair, and consequently was moved from one train to another in comparative comfort, but at Bath station none but an office chair could be obtained, and she was carried out of the station on it. Not being a newly-elected member of Parliament, nor the captain of a victorious football or cricket team, it is possible that she found the honour of being "chaired" trying; and the incident will hardly encourage crippled invalids to seek relief from their sufferings where such honours await them.

### WANTED A LIMIT TO EXAMINATIONS.

How difficult are nursing examinations to become? Anatomy, physiology, pharmacy, hygiene, dressing midwifery, the care of infants, are all included. Candidates are also expected to discourse learnedly on the "constitutents of the blood" and "the revolution in the treatment of hospital patients," and kindred subjects. Amidst so much intellectual achievement, which after all belongs more to the physician and surgeon than to the nurse, is there not considerable danger of crowding out more homely, but more important from a nurse's standpoint, duties? We think so.

### DUTY IN SPITE OF THE SHOUTS OF FIFTY MEN.

MEN are not always ready to welcome lady coadjutors on the Board of Guardians, for women have not always the necessary knowledge of business routine nor the tact to overcome hostility; consequently, a scene such as took place at a meeting of the Rochford Guardians is possible, although it is the reverse of creditable to any concerned in it. A committee was appointed to select and recommend a master, matron and head nurse of the workhouse. Mrs. Seel was the only lady present at the meeting of this committee. The gentlemen retired into another room, without the lady, "to discuss" matters, according to their own account, to hold a "secret committee," according to Mrs. Seel's; with the result that, according to this lady's statement, the new appointments were made without reference to her. When the question of secret committees was raised at a meeting of the Board, it w a treated with ridicule. Mrs. Seel was excused jocosely on the score of "curiosity being a feminine failing," until, amidst "roars of laughter," she announced her intention of doing her duty, as she did not mind being shouted down by fifty men. Mrs. Seel is evidently not one of the "door-mat" kind according to *Punch*. We fear that her way of handling the matter made it easy for her opponents to raise a laugh against her, but there is reason in her contention. She has been chosen to represent the ratepayers in the management of a parochial institution, and it is certainly her duty to inquire into the appointment and qualifications of important officials, and to take her share in the decision of any committee on which she sits; therefore, the men Guardians had no right to exclude her when such matters were discussed, they had no right to sneer at her curiosity on such points, and they certainly did not show that courtesy (unfortunately so often absent from public meetings) which, when displayed towards an opponent, robs opposition of bitterness and forwards progress.

### IF FOR MILK, WHY NOT FOR WAGES?

MOST of the troubles arising in workhouses anent the nursing spring from a want of good management. The constant changing of nurses, such as was complained of lately by the Doncaster Board of Guardians, reflects little credit on any concerned, and the reasons given for it in this instance are certainly instructive. One Guardian laid the blame on the old workhouses, and suggested that as some members advocated an increase in the price of milk they should also advocate an increase in the nurses' salaries. Another remarked that he did not believe in sweating one class of the employed and overpaying others, and said the salaries should be reorganised throughout. A good superintendent nurse, well backed up by her committee, would soon mend matters.

### OUTDOOR PAUPERS.

A RESOLUTION in favour of appointing a committee to inquire into the desirability of securing a nurse to attend to outdoor sick paupers, subject to the approval of the Local Government Board, was lost at a recent meeting of the Farnham District Nurses' Association. This point will certainly come forward again, as it concerns a difficulty frequently arising between the Guardians and district nursing associations. District nurses are often called upon to attend outdoor paupers, and Guardians sometimes do, and sometimes do not, recognise a pecuniary obligation in the matter. When they do they subscribe to the nursing fund; when they do not, they keep the money.

### THE PANACEA OF AN IMPRESSIONIST.

"THE less one knows about a subject, the more interesting it is to write about." This is the avowed principle that guides a certain class of journalists, and which certainly produces amusing "copy." An irresponsible correspondent of this class, writing to a society journal, disposes of some of the difficulties that beset the nursing world in this rough and ready way, and it is not impossible that readers as thoughtless as himself will accept his statements as practical solutions of questions that involve wide issues. For instance, most people are aware by this time that a certain number of probationers seek work at the training schools for many other reasons than the apparent one, viz., in order to become nurses; also that troubles do occasionally arise from young and attractive women nursing young men. This correspondent strikes down both evils with one blow. He says, "Let no woman under the age of 30 nurse a male patient either in the wards of a hospital or outside; their places can be better occupied by men." He aims another decisive blow when he proposes to cut down fees to 20s. a week and board as the outside limit; and he finally finishes the unqualified midwife by proposing she shall be tried for manslaughter for every death amongst her patients. His drastic remedies would doubtless put an end to the diseases, but unless he found a panacea for all the ills that human flesh is heir to in the meantime, the sick will have a sorry time of it, for there would be no trained nurses, and Sairey and her compeers would once more reign.

### SHORT ITEMS.

LAST March Miss Peter, Queen Victoria's Jubilee Institution, visited Rushden Nursing Association and gave a thoroughly satisfactory report. The association has begun its seventh year's work, and has Nurses Boge and Pritchard in its service.—Nurse Wallis, of the Gravesend Hospital staff, has commenced work as district nurse at Northfleet, in connection with the Samaritan Fund. This is the Jubilee memorial of the parish.—The Cheadle and Gatley nurse, Miss Smethurst, has worked very hard last year. For the first time since the foundation of the district association seven years ago the balance is adverse. The expenses have been much the same, but the support of many members has been lost through death. Mr. Hardcastle Sykes generously cancelled the debt to the treasurer, and the association starts fair once more.—In honour of the year the Liverpool Needlework Guild has sent the Queen Victoria District Nursing Association 300 garments for distribution in the newly-established nursing districts.

### Practical Aspects of a Nurse's Life.

By Sister Grace.

### X.—PROBATIONERS IN SURGICAL WARDS.

### Operations on the Urinary Organs.

Operations for the removal of stone from the kidney, or for the removal of any growth from the kidney, resemble, to a great degree, many other operations, and therefore I must not speak much of them to-day as my space is limited; but I may as well tell you that in all such operations you will be expected to measure the urine passed, and to keep specimens daily.

### Lithotomy is the operation usually resorted to for the removal of stone from the bladder. Lithority, even if feasible, is an irritating operation, because if the operator has been successful in crushing the stone, there still remains the process of removing the fragments, the larger probably by forceps, and the smaller by the slow and painful washing away by the flow of urine. By lithotomy, that is to say, immediately cutting down upon the stone, the operation, if successful, gives speedy relief, and the ability to eat and drink as usual within a short time makes the patient feel on a rapid road to recovery. The operation is usually supra pubic; an incision will be made, large enough to allow the passage of the stone, directly into the bladder above the pubes. You will notice that the sister has an apparatus at hand to measure the quantity of fluid injected into the bladder before the operation commences. It will probably be some time before you are yourself called upon to make these preparations for serious operations; but keep your eyes open and see what is used, and if your sister takes interest in her probationers she will be ready to explain things to you at suitable times; but do not run into the error of teazing her with questions when she is much occupied, and when you ought to be busy with ward work.

Probationers are often ill-advised in this respect, and then complain that the sister and staff nurse take no trouble to teach them. When they are very busy, and their thoughts occupied with a serious operation which will cause the patient's life to tremble in the balance, you can see that it is not a propitious moment for a probationer to seek for information; but if you are a hard-working woman, and take interest in your work, your sister will find the opportunity to explain the necessary preparations to be made, and the different course of treatment and nursing to be observed in the several stages towards recovery.

I once had a probationer of great intelligence who passed all her examinations brilliantly, but who lacked the practical qualities that make a good nurse; amongst other wants she had no "tact," which, I think, may be otherwise translated thus: she had not natural good feeling and courtesy, for that is really the foundation of tact. I remember one day I was undressing a man brought in from the railway with both legs off, whose collapsed condition was so serious that it was a question whether it was possible to take him to the theatre; this was the suitable moment my nurse chose to ask, "Oh! sister, now you will be able to explain to me what I never can understand. When a large artery is severed, how is the circulation continued?" As may be imagined, my feelings to her were not very amiable, and I replied somewhat sternly, "At present our duty is to try and save a life, not to study the circulation; occupy yourself in removing these soiled clothes." A woman who, in the face of such a tragedy, could seek a clinical lecture on the circulation, would never make a nurse as long as the sun and moon endures.

To return to the patient. After operations of this nature, either for stone or malignant growth, to keep the bed even comparatively dry is a difficult problem. Probably at first drainage will be arranged by means of a long tube introduced into the wound, but as a rule this plan is not continued long, on account of the tendency to keep the wound open, and so to retard the flow of urine by the natural passage. This will take place, according to circumstances, from a fortnight to a month after the operation, and you must prepare accordingly; the many elaborate and expensive mechanical appliances invented to combat the intervening time of discomfort are none of them to my mind wholly satisfactory. I think good macintoshes and frequent changes are the best arrangement. It is most important to keep the skin from being chafed by the constant dribbling of urine; each time the dressing is changed, which will be about every three hours, smear the surrounding parts well with antiseptic ointment; this, combined with frequent washing, will keep your patient as comfortable as circumstances permit.

### Perineal Section is undertaken for the removal of stone from the urethra, for certain cases of stricture, and also when the urethra has been ruptured from an accident, also for extravasation of urine, &c. If it has been performed for impacted calculus or for extravasation, the effect as to personal discomfort is the same as in the supra-pubic operation. There is in all these cases a great tendency to rigors; watch the patient closely, and at the first symptom wrap him in hot blankets and take his temperature, which will probably be very high. All sufferers from urinary disorders are liable to rigors; you will notice it frequently in cases of stricture, always common in the male wards of any hospital. The rise of temperature is often so sudden and excessive as to tax one's powers of belief. I remember a singular case of internal urethrotomy which had done well for three days; suddenly a rigor took place, the thermometer registering 107·4. I could not believe my own eyes, and took the temperature with a second instrument, obtaining, however, the same result. The man became slightly delirious, but at the end of twelve hours had returned to his former state, and made a rapid recovery. I have necessarily confined myself to speaking only of those operations which you will see most frequently in the hospitals; the same rules for strict obedience to orders and devotion to your patients must actuate you in all cases, and though treatment and symptoms differ, the same conscientious care is required in all.

### Minor Appointments.

The Eastern Fever Hospital, Homerton.—Trained at the Nightingale Fund School and at St. Thomas' Hospital, ward sister and night superintendent at Radcliffe Infirmary, Oxford, and latterly sister-housekeeper at Charing Cross Hospital, Miss E. M. Littlewood is well qualified for her new appointment as Assistant Matron at the above institution.

Royal Hospital for Sick Children, Edinburgh.—Miss Amelia Day is the recently-appointed Assistant Matron of the above. She was trained at the Batley District and the Sheffield Royal Hospitals, and has been night sister and ward superintendent at Sheffield.

Children's Hospital, Queen Street, Belfast.—Miss Eveline A. Stanley, trained at West Bromwich District Hospital, Staffordshire, is the new Sister here. She has also been staff nurse at the Royal Infirmary, Derby.

### Post-Graduate Clinics for Nurses.

By a Trained Nurse.

XXXVI.—THE NURSING OF TYPHOID (*continued*).

It will be well, in order to make the feeding of a typhoid fever patient more intelligible, to trace briefly the condition of the intestinal tract during the progress of the fever.

The stages of the disease when once this has declared itself are marked in the following manner. During the first week the glands, the so-called "solitary" glands scattered through the whole of the large and small intestine, and Peyer's patches in the lower half of the small intestine, especially in that portion passing into the large bowel, become inflamed and swollen. In the second week in a mild and favourable case—and this is common enough in children —the inflammation subsides. But in a bad case the inflammation goes on to sloughing, and in the third week the sloughs separate, leaving ulcers, superficial or deep, according to the seriousness of the case. In the fourth week in a normal case the ulcers show signs of healing.

During the first two weeks slight hæmorrhage from the bowel is not necessarily a bad sign. Indeed, some doctors regard it as a favourable symptom. For it must be remembered that hæmorrhage in the early stages arises from congestion of the mucous membrane. Arising in the third week it is a grave symptom, since it indicates ulceration into a blood vessel. And the patient by this time is so exhausted from continued fever that he cannot stand further drain on his strength. The nurse must carefully watch her patient, for in the third week, if constipation be present, the patient may lose a serious amount of blood before this shows externally. Sudden pallor, fall of temperature, and chilliness of patient are symptoms of this condition, and hæmorrhage occurs in from 7 to 10 per cent. of typhoid cases. The nurse, always on the watch for blood in the motions, should communicate at once with the doctor if this appears in the third week, and should apply ice-cloths abdominally. Vomiting sometimes sets in at the same time as the hæmorrhage—often arising from exhaustion—when the nurse should apply a mustard poultice to the epigastrium, give nothing by the mouth but ice to be swallowed in pieces, and administer small nutrient enemata, remembering always in typhoid, if the lower bowel be irritable, that soaking the enema tube in very hot water for five minutes beforehand often enables the enemata to be given without rejection. In severe hæmorrhage the foot of the bedstead should be raised, and every precaution taken to ensure the utmost rest and quietude. Perforation of the bowel—if this take place—will occur either in the third or fourth week, owing to the bed of an ulcer tearing through the intestines. Collapse, severe abdominal pain and rigors will mark this condition, followed by distension and peritonitis. It must not be forgotten that in enteritis the mucous membrane of the stomach is often involved in the general inflammation, rendering the stomach very irritable, and causing a deficiency in the normal digestive secretions. The exact digestive function of the intestinal glands involved is not quite understood, so that the great differences in digestive disturbance—in some cases of typhoid so slight, in others so severe—is not accounted for.

The typhoid temperature, so characteristic, increases steadily during the first week, varying with the extent of the intestinal inflammation. The type of the temperature constitutes the "remittent" nature of the fever, falling in the morning and rising each night to a higher point till it reaches 103 deg. or 104 deg., or still higher points.

In the second week the fever is continuous, and in the third week again assumes the remittent type, falling towards morning and rising again at night. In mild cases the fever subsides in the third week, and each morning shows a lowering in the chart. In severe cases it remains high, and may be continuous instead of remittent, even in the fourth week.

The treatment for the high fever varies with the physician in charge. Cold sponging may be ordered, in which case the patient between blankets is sponged with tepid, cold, or ice water, to be continued till the temperature is brought down to a given point.

Some doctors order the cold bath; but, of course, neither this nor cold sponging or wet packs must be given without medical instructions. In cold bath treatment the patient is lowered into a bed-side bath of water at 95 deg., cooled gradually with ice or cold water down to 70 deg.; or another method is to immerse the patient for 15 minutes in water at 65 deg.

Many nurses say that "typhoids are uninteresting patients but interesting cases." The comatose apathy and depression accompanying this disease necessarily make the sick-room very quiet and free from excitement. But the good nurse sees in a typhoid case that opportunity to be eyes, hands, and second self to her patient, which is most "interesting."

In early stages the nurse must remember that her patient has a long, feverish time to pass through, and that every effort must be made to keep his strength in "trim" to fight against the exhaustion of the disease. Lung and throat complications may easily ensue in a mild case through chill after sponging or through insufficient clothing of the patient. The bed, too, needs scrupulously guarding from draught. By change of position the nurse may minimise the risk of lung trouble, for continued lying on the back intensifies the tendency to bronchitis and pneumonia. Many nurses think lung trouble is caused by remaining too long in the same position, but this is erroneous, since position is another factor added to a tendency and is not in itself a cause.

The patient should be sponged night and morning with tepid water. The unpleasant odour frequently arising from the body of a typhoid patient may be counteracted in prosperous households by the addition of a small quantity of eau de Cologne to the bath water. To minimise the risk of chill and refresh the feverish skin it is well to give the patient a gentle "rub down" after the sponging, especially over the chest and back, with hands saturated with Cologne water. Methylated spirit may be used where the expense of Cologne might be a consideration. Children's hair should be cut short from the first day of typhoid. Women patients naturally hesitate to sacrifice their hair. But this must be done in bad cases, and often where delirium and high fever supervene the head must be shaved to allow of the necessary evaporating lotions, ice-bags, or cold compresses to the head. Cutting the hair off is the best step in all severe cases of typhoid, as it will otherwise fall out and grow thin and scanty.

The condition of the mouth and throat is most important, since the hygienic condition of these materially affects the feeding of the patient. An admirable mouth wash consists of glycerine, lemon juice, rose water, and chlorate of potash solution in equal proportions. Boro-glyceride diluted with water may be used for the tongue and throat, applied with a soft brush or feather.

### Wants and Workers.

"F. M. R.," 36, Mortimer Street, Langham Place, W., wishes to know of a home or family to which a gentleman, somewhat delicate, could be admitted on low terms. Hastings or neighbourhood preferred.

Can any of your readers tell the Matron, Children's Cottage Convalescent Hospital, Wokefield, Mortimer, Berks, of a home where she can place a girl of 10 who has had diseased bone in leg and also diseased spine? She is able to get about, but wears a support for the leg and another for the spine. The parents are poor, but can give small weekly payments. Full particulars of the case will most gladly be given.

### Our American Letter.

American nurses are in arms against a growing tendency they imagine they perceive amongst their own ranks. It appears that the first National Association for American nurses has for its president a British subject, and there are not wanted nurses who declare that as the President of the United States must be a natural-born citizen, the same rule should hold good throughout. The nursing papers are also catering too much for the English press in the opinion of some, and the cry comes for American nurses to be more American and to shut out rigorously English nurses and English ideas, and to do their own nursing in their own way, and to think their own thoughts upon this subject.

Free trade is too wide a subject for nursing. Political economy is for the politician, but surely the teacher should instruct himself (or herself) first in superior schools before mounting the professor's chair. Our English nurses should note this unfriendly attitude towards outside influence by the American members of the fraternity of nurse-craft. Often English nurses turn their eyes to America, hoping to see a wider field of usefulness opening there for them, but intendant emigrants who contemplate leaving England to make their homes across the Atlantic should know they are going among those who strongly object to entertaining strangers, even though they may be angels unawares.

The graduates of the training school, New York, are determined to have a Nurses' Club, and have arranged a hospital fair in November in the administration building of the New York Hospital, in order to raise the money. Stalls will be spread with dainties, nick-nacks, embroideries, and needlework, and music and entertainments will be the order of the day. It is the intention of the promoters of the club to hire a house; the lower part will be furnished as dining, sitting, writing, and club rooms, and the upper part let to nurses room by room as it is wanted.

The administration block of the hospital in which the fair is to be held is of historic interest. It was formerly known as Thorne Mansion, and was one of the early homes of New York; the rooms are adorned with tapestry and old pictures, and some of the oak carving is very fine. The days, however, of the picturesque old house are numbered, for it must be pulled down to make way for a more convenient if not so imposing a structure.

There is plenty of activity in hospital building just now in America. About 100,000 dollars is to be spent in construction at Rhode Island Hospital, at Collins' Farm Hospital, Buffalo, New York, improvements costing 300,000 dollars are in progress, a new ward is to be added to Eastern Maine General Hospital at Bangor, and an emergency hospital is to be built in the town of Chicago before winter sets in. The new wing at Rhode Island Hospital will have 100 patients. There will be wards for cancer, gynæcology, children, male surgery, and eye and ear work for both men and women. The building will be of brick, three stories high, and will probably communicate with the block already standing by a covered way.

The hospital at Collins' Farm will probably be one of the finest in the State when finished. One hundred and thirty beds will be added. They will be in wards, single rooms, and dormitories. The building and fittings are to be of the latest modern invention. A kitchen, bakery, and store rooms will be placed in a separate block, as will also a laundry. Steel seems to enter largely into the construction. The roofing of the commissariat department is to be of steel, and there is to be a steel tower attached to the power house. Precautions are taken to render the new hospital practically fireproof.

### Centenary Celebration at the Royal Infirmary, Sheffield.

There was quite a transformation scene here on Friday afternoon, October 29th, when, in response to a widespread invitation, the citizens of this city turned out to view the new buildings, which have been erected at a cost of £20,000. They were received by the Duke of Norfolk, president of the institution, and after a few preliminaries in the form of speeches dealing with the purpose of each department, Lady Mary Howard was asked to declare the Nurses Centenary House open. This is a large stone building, with ample accommodation for sixty nurses. The ophthalmic department and operating theatre proved specially attractive, care having been taken to bring them as far as possible up to the modern standard of excellence. The scenes were brilliant, many ladies being present, and the band of the York and Lancaster Regiment providing inspiriting music. Many of the guests visited the wards of the parent building, and were agreeably surprised at their cheerful look. The children's ward was thronged, and great interest was shown in the small patients.

The Duke of Norfolk presided over the banquet held the night before, and Sir W. McCormac, P.R.C.S., and Sir W. Broadbent were among the guests.

A fitting and happy conclusion to the more ceremonious proceedings was an enjoyable dance, in the evening, at the Cutlers' Hall, where the nursing staff were to be seen in their pleasant uniforms making the most of the opportunity afforded them of a thorough relaxation.

None the less did the patients appear to enjoy themselves on the evening of the 5th at an impromptu concert and entertainment got up for their special benefit.

A handsomely bound and profusely illustrated volume dealing with the history of the institution by Mr. J. D. Leader, and a biographical sketch of the past honorary staff by Mr. Simeon Snell, has been published as a memento of the hundred years of work of the institution.

### Where to Go.

British Home for Incurables, Crown Lane, Streatham, S.W.—On December 2nd, at 2.30 p.m., a sale of patients' work will be opened by Mr. J. Hampton Hale. The proceeds are for the patients' Christmas entertainment. Admission free.

St. Mary's Hospital.—A concert will be held in aid of St. Mary's Hospital at the U.M.F.C. Hall, Queen's Road, Bayswater, on November 24th, at 8 p.m. Many artistes have promised their services.

Victoria Commemoration Club, 29, Southampton Street, Strand.—A lecture on "The Goldfields of the Yukon" (Klondyke) will be given by Madame Alting-Mees on December 2nd, at 3 p.m.

### Novelties for Nurses.

### A SOAP FOR THE HAIR.

We have received a sample of a very nice soap from Messrs. Peacocks, 14, Queen Victoria-street, which we are glad to draw our readers' attention to because it helps to economy of time and money. The use of egg in cleansing the hair is very popular, but it is attended with many drawbacks which we need not point out. It is claimed by the manufacterer that "Ovaline" soap is really made of eggs, amongst other ingredients; it is pleasant in use, nicely scented, and well made. It forms an excellent lather, and is very softening to the skin.

### Appointments.

### MATRONS.

Chertsey and Ottershaw Fever Hospital.—Miss Clara A. Ford has been appointed Matron and Superintendent of Nurses of this hospital. Her career is three and a half years probationer at Chichester Hospital, two years sister of medical and surgical ward at the Devon and Exeter Hospital, Exeter, and four and a half years night superintendent of the same.

### For Reading to the Sick.

"LO, I AM WITH YOU ALWAY."

—*S. Matt. xxviii.*, 20.

**Verses.**

FOR the love of God is broader
Than the measure of man's mind;
And the Heart of the Eternal
Is most wonderfully kind.
But we make His love too narrow
By false limits of our own;
And we magnify his strictness
With a zeal He will not own. —*Faber.*

O thou that in our bosom's shrine
Dost dwell, unknown, because divine!
I thought to speak, I thought to say
"The light is here; behold the way."
"The voice was thus," and "thus the word."
And "thus I saw," and "thus I heard."
But from the lips that half essayed
The imperfect utterance fell unmade,
Unseen, secure in that high shrine,
Acknowledged, present and divine;
I will not ask some upper air
Some future day to place thee there.
Do only thou in that dim shrine,
Acknowledged present and divine;
Be thou but there! In soul and heart.
I will not ask to feel thou art. —*Clough.*

Oh, Heart! weak follower of the weak,
That thou should'st travel land and sea
In this far place that God to seek
Who long ago had come to thee.
—*Lord Houghton.*

O, great good God, my prayer is to neglect
The shows of phantasy, and turn myself
To Thy unfenced, unmeasured warmth and light!
Then were all shows of things a part of truth;
Then were my soul, if busy or at rest
Residing in the House of perfect Peace.
—*Allingham.*

Change and decay in all around I see,
O, thou who changest not, abide with me. —*Lyte.*

I was not called to walk alone,
To clothe myself with love and light;
And for Thy glory, not my own,
My soul is precious in thy sight.
My evil heart can never be
A home, a heritage for me;
But thou can'st make it fit for Thee.

**Reading.**

"Lo, I am with you alway! I am with you to succour in temptation, to strengthen in duty, to guide in perplexity, to comfort in sorrow. From the instant you become a disciple I am with you all along. All your life I am with you—and at death? At death, you are with me: that is the difference."—*Mount of Olives.*

"Admit Him into your home, that He may hallow it. Admit Him into your hourly occupations, that He may elevate and expedite them. Admit Him into your happy moments, that He may enhance them; and into your hours of anguish, that His presence may tranquilize and transform them. Let His recollected presence be the brightness of every landscape, the zest of every pleasure, the energy of every undertaking, the refuge from every danger, the solace in every sorrow, the asylum of your hidden life, and the constant Sabbath of your soul. Learn to have—not one life for God and another for the world, but let your earthly life be divinely directed and divinely quickened, and let every footstep be a walk with God."

### Notes and Queries.

**The contents of the Editor's Letter-box have now reached such unwieldy proportions that it has become necessary to establish a hard and fast rule regarding Answers to Correspondents. In future, all questions requiring replies will continue to be answered in this column without any fee. If an answer is required by letter, a fee of half-a-crown must be enclosed with the note containing the enquiry. We are always pleased to help our numerous correspondents to the fullest extent, and we can trust them to sympathise in the overwhelming amount of writing which makes the new rules a necessity. Every communication must be accompanied by the writer's name and address, otherwise it will receive no attention.**

*Companionship.*

(60) You kindly inserted a query in your paper for me lately. May I trouble you still further? I am anxious to meet with another nurse, like myself, studiously inclined, who would accompany me to the Royal College of Surgeons, and, perhaps, afterwards to other lectures and nursing exhibits. I know several nurses, but they are all more or less giddy and dressy. I want to meet one who loves work like myself. We would each pay our own expenses.—*A Fellow Student.*

The Victoria Commemoration Club, 28 & 29, Southampton Street, is just what you want. The membership is £1 1s. per annum, and you would meet there the studious and thoughtful nurses whose companionship you desire. Write direct to the secretary.

*Therapeutics.*

(61) Will you please recommend to me a book: the names of medicines, with their properties and effect. I should also be glad if any of your readers could give me a cure for flatulence, as I have a patient who suffers so much, and can find no effectual remedy.

You will find "Materia Medica and Therapeutics," by J. Mitchell Bruce, M.D. (Cassell and Co.), 7s. 6d., handy and full. It is trenching on the medical man's province to suggest remedies. Have you called your patient's doctor's attention to her flatulence? You seem to have a taste for medicine, but before you can gratify it you should become a doctor. In our queries lately we gave a list of the medical schools for women.

*Prayers for Hospital Use.*

(62) In reply to the inquiry for a suitable book of prayers for hospital use, a Provincial Matron finds the one she has used for some years past always appreciated by the patients. It is "The Hospital Prayer Book," arranged by Edward John Waring, M.D., published by J. and A. Churchill, 7, Great Marlborough Street.

*Training.*

(63) Could you very kindly tell me if a certificate for two years' general training in a hospital of over 100 beds, and then another year as staff nurse in a different hospital would count as three years' training? I am a nurse, and find that so few posts are open to me without three years' training.

Two years' training and one year in a large infirmary as staff nurse would in some instances be more valuable than three years' training in one place, but it does not count as three years' training. We should imagine that your training and experience will admit you to good posts, especially where the appointing authority understands nursing matters.

*Homœopathic Nursing.*

(64) 1. Would you please to tell me, through your paper, where the best homœopathic hospitals are? 2. Would I receive a good training in one of these without paying a premium? 3. Would any more be required from me there than at any other hospital?—*E. B.*

Apply the Lady Superintendent, London Homœopathic Hospital, Great Ormond Street, W.C., or the Lady Superintendent, The Hahnemann Hospital, Liverpool. You would receive a good training in either, and nothing more would be expected of you than in any general hospital. The lady superintendent will inform you when a vacancy occurs if you write and ask; also what advantages, if any, are granted paying probationers.

*A Year's Training.*

(65) Will you kindly tell me the names of hospitals (if there are any) where probationers are received for a year's training without premium, and where a small salary is also given?—*R. H. M.*

There are one or two hospitals—Stockton and Thornaby is one—which take probationers on such terms. "How to Become a Nurse," H. Morten (2s. 6d., 28 & 29, Southampton Street, Strand), gives a list of all training schools, which you should consult. Let us, however, urge you, if you put your hand to the nursing plough, to train thoroughly. If you take a three-year course you would be paid from the first in some excellent schools.

(66) 1. Will you kindly inform me through the medium of your paper of a good, useful dictionary for a nurse and price of same? and what is the price for binding a volume of THE HOSPITAL and "Mirror"? and oblige.—*Wembdon.*

"Hoblyn's Dictionary of Medical Terms," 10s. 6d., Whittaker and Co., Paternoster Row. (2) Binding THE HOSPITAL costs 2s. 6d. at the publishing office.

*Master or Patient.*

(67) Will you kindly tell me whether it would be correct for the friends of a patient to say to a trained nurse, private nursing, "master and mistress" or "your patient"? I know resident governesses do not come under the same heading as domestic servants; do hospital-trained nurses?—*Nurse W.*

"Your patient" is the usual mode. The nurse is under the command of the medical attendant. The patient certainly employs her, but is not therefore either master or mistress.

